# A Special Section of Selected Reviews From the Plant & Animal Genome XII Conference

**DOI:** 10.1002/cfg.400

**Published:** 2004-04


					Comparative and Functional Genomics
A Special Section of selected reviews from the
Plant & Animal Genome XII Conference
10-14 January 2004, San Diego, California
I
N
T
E
R
N
A
T
I
O
N
AL PLANT & ANI
M
A
L
G
E
N
O
M
E
CONFERENCE
For free access to the articles, visit:
http://www.interscience.wiley.com/journal/cfg -- the CFG website (HTML and PDF formats)
http://www.intl-pag.org/ -- the PAG website (PDF format)
Plant Genome I was held 12 years ago in November 1992. In January 1997, the conference expanded
its remit (to include livestock), becoming Plant and Animal Genome V. The conference has included
plant and animal genome sessions every year since then. Each year the attendee numbers grow and at
least one research group breaks the news of a significant achievement at the conference, indicating its
importance to each of the communities holding workshops there. Abstracts from all of the past meetings
are available for searching from the PAG website: http://www.intl-pag.org/
Organizing Committee
Chair: Stephen R. Heller, NIST, USA
Co-Chairs:
Plants: Animals:
Rebecca Doerge, Purdue University, USA Bhanu Chowdhary, Texas A&M University, USA
Michael Gale, John Innes Centre, UK Noelle Cockett, Utah State University, USA
Ed Kaleikau, USDA/CSREES, USA Catherine Ernst, Michigan State University, USA
Dave Matthews, USDA ARS, Martien Groenen, Wageningen University,
Cornell University, USA The Netherlands
Graham Moore, John Innes Centre, UK
Jerome P. Miksche, Emeritus Director, USDA
Plant Genome Program, USA
Rod Wing, Clemson University, USA
Conference Organiser:
Scherago International, 11 Penn Plaza,
Ste 1003, New York, NY 10001, USA
http://www.scherago.com
PAG logo reproduced by permission of International Plant & Animal Genome Conference

				

